# Green Open Innovation Activities and Green Co-Innovation Performance in Taiwan’s Manufacturing Sector

**DOI:** 10.3390/ijerph17186677

**Published:** 2020-09-14

**Authors:** Ching-Hsun Chang

**Affiliations:** Department of Technology Application and Human Resource Development, National Taiwan Normal University, Taipei 106, Taiwan; chang.ch@ntnu.edu.tw

**Keywords:** corporate sustainability, appropriability regime, perceived similarity, green open innovation activities, green co-innovation performance

## Abstract

This study investigates the positive effects of an appropriability regime and perceived similarity to green co-innovation performance when considering green open innovation activities as the mediator. It proposes a novel construct, i.e., green open innovation activities, and employs structural equation modeling to test its hypotheses. 190 valid questionnaires were collected from executives in Taiwanese manufacturing companies. Manufacturing activities are regarded as a major source of pollution. Consequently, given the broad concern for the environment among governments and consumers, adopting green practices has become critical for manufacturing companies. All the proposed hypotheses were supported by the analysis results. An appropriability regime is positively associated with green open innovation activities and green co-innovation performance. Perceived similarity is positively associated with green open innovation activities and green co-innovation performance. Moreover, green open innovation activities are positively associated with green co-innovation performance. A major finding is that if a company introduces one, the longer the duration of a green project is, the stronger the green open innovation activities and green co-innovation performance are. This study aimed to determine the simultaneous effects of both factors, i.e., appropriability regime, and perceived similarity on green open innovation activities and green co-innovation performance. The contribution of this study highlights the simultaneous importance of appropriability regimes and perceived similarity to determine a company’s green practices. While companies have tended to increase their green co-innovation performance, they need to improve their appropriability regime, perceived similarity, and green open innovation activities.

## 1. Introduction

The United Nations proposed its Sustainable Development Goals (SDGs) in 2015. The 17 goals aim to achieve economic growth and social advancement in a sustainable manner that ensures that the environment is protected. Different from the United Nations Millennium Declaration (MDG), SDGs include a vision to establish systematic partnerships with the private sector to achieve sustainable development. SDGs provide guidance for companies’ daily operations. Companies cannot attain SDGs on their own. They have to transform their knowledge with inside or outside partners [1]. The green co-innovation performance of companies is triggered by customers’ or business partners’ expectations. For example, following the trend of environmentalism, Apple is committing to total carbon neutrality by 2030. Taiwan Semiconductor Manufacturing (TSMC) has become the world’s first semiconductor company committed to 100% renewable energy usage by 2050. Nike has developed the Supply Chain Sustainability Index (SCSI), which sets clear and consistent minimum sustainability requirements for logistics service providers. Nike will evaluate suppliers’ efforts in sustainable development in response to customers’ expectations. Many companies have sought to integrate these goals into their projects to ensure compliance with various regulatory regimes and to inspire the trust and support of consumers. This study investigated Taiwanese manufacturing companies because manufacturing activities are regarded as a major source of pollution. Consequently, given the broad concern for the environment among governments and consumers, adopting green practices has become critical for manufacturing companies. In particular, various stakeholders tend to be concerned about the eco-friendliness of the practices of companies as well as their environmental stewardship. Therefore, SDGs provide a vision for companies that can align their strategies as well as manage their contribution to the realization of green co-innovation performance.

Companies now tend to consider environmental factors in their operations and strive for green innovation. However, a single company’s development of green products requires the devotion of considerable time for research and incurs substantial costs; typically, such development cannot be accomplished using the internal resources of a single company. Instead, companies must cooperate with external partners to take advantage of knowledge from various fields. This enables companies to generate creative ideas for reducing costs and waste during product or process development with their business partners, which results in improved operational performance. Given the value of partnerships in developing sustainable practices, this study focuses on green open innovation activities and green co-innovation performance. If a company can successfully achieve green innovation, it can promote its commitment to sustainable development and encourage consumers’ sustainable consumption.

Open innovation and co-innovation have been widely discussed in the literature and implemented in practice [2,3,4,5,6]. To improve green open innovation activities and green co-innovation performance, companies must develop both factors. One is a company’s appropriability regime, which is defined as the extent to which companies can manage internal knowledge and protect it from appropriation by competitors [7]. The other factor is perceived similarity, which refers to partners sharing a similar work ethic or common cultural background [8,9]. Sharing information among partners can facilitate and expedite innovation success [10]. Few studies, however, have examined both factors in the context of sustainable innovation. Thus, this study aimed to close this gap in the literature.

## 2. Literature Review and Hypotheses Development

### 2.1. Green Open Innovation Activities

Open innovation refers to companies using both their internal capabilities and those from external sources to create valuable innovations [7,8]. Open innovation may occur in one of three forms [9]. In outside-in open innovation, a company incorporates a variety of external influences into its innovation processes. By contrast, if a company adopts an inside-out approach, it may permit its idle or unused ideas to be used in the business models of other firms. The final approach is coupled open innovation which is a combination of the preceding two [10]. Through open innovation activities, companies can integrate inside and outside knowledge with their business partners.

A firm can acquire a sustainable competitive advantage through green product and process innovation. Based on the definition of “open innovation activities” [11], in this study the idea of green open innovation activities is developed; this refers to a process whereby companies combine internal research with external knowledge of sustainable development to create innovations. According to stakeholder theory, value creation is a collaborative activity in relationships between the focal company and its external and internal partners, such as customers, end-users, suppliers, shareholders, government, and business partners [12,13]. This study aims to verify green open innovation as a mediator and offers a new perspective on both factors in the context of sustainable innovation.

### 2.2. Hypothesis Development

#### 2.2.1. Positive Effect of Appropriability Regime on Green Open Innovation Activities and Green Co-Innovation Performance

Sustainable development depends on successful green innovation. To protect their innovations, green or otherwise, companies adopt measures that safeguard intangible assets. Intellectual property rights (IPRs) can be protected through filing patents, receiving a copyright or trademark for a product, or designating proprietary information as trade secrets and suing those who would seek to exploit such information [14]. The aforementioned measures constitute the appropriability regime of a company, which enables it to protect its internal knowledge [7]. In addition to legal tools, a company may adopt various human resource management policies or practical or technical means of preventing appropriation. Appropriability mechanisms protect IPRs, and in providing such protection, they can also serve as a deterrent or barrier to rivals acquiring a stronger competitive position. For example, a patent protects intellectual property, and the very nature of a patent is to reward innovation with exclusive rights to exploit this innovation. Having a strong appropriability regime can ensure cooperation without leaving a firm’s competitive position highly vulnerable to competitors.

As noted, a company may adopt various green open innovation types. Outside-in green activities entail a company obtaining knowledge from external sources when they do not possess environmental knowledge and capabilities to create green innovations of their own. Companies obtain environmental management knowledge from outside parties, such as marketing consultants, universities, and other research institutes. In this manner, companies can develop ideas for future green innovations. Inside-out green activities involve the development of environmental knowledge and new technology internally and then sharing developed innovations with partners [15]. Through research and development (R&D), companies can develop and enhance environmental management knowledge acquired from outside the firm. In a coupled process, a company engages in both inside-out and outside-in innovation [11].

Co-innovation entails integrating internal and external knowledge to generate new products or services [8]. To effectively do so, companies must establish mechanisms to integrate various resources, whether internally generated or sourced from outside [16]. When companies have appropriability mechanisms to protect patents from competitors, this can help companies to gain, create, and transfer knowledge and resources within the focal company and beyond its boundaries. An appropriability regime allows the focal company to decide how to execute protective power for controlling green innovations. An innovating company has a hard time fully benefiting from its patents or copyrights commercially [12]. If the company can collaborate on green innovations with its business partners, an appropriability regime can promote co-innovation under the trend of environmentalism by allowing new combinations of innovation. In summary, a strong appropriability regime can protect the current knowledge of companies, facilitate green open innovation activities, and improve green co-innovation performance. Hence, the following hypotheses are proposed:

**Hypothesis** (**H1**)**.** *An appropriability regime is positively associated with green open innovation activities.*

**Hypothesis** (**H2**)**.** *An appropriability regime is positively associated with green co-innovation performance.*

#### 2.2.2. Green Open Innovation Activities have a Positive Effect on Green Co-Innovation Performance

Co-innovation performance describes the effectiveness of a company’s synthesis of internal and external ideas to create a platform to generate new revenue [17]. In co-innovation, companies collaborate with particular firms that can satisfy particular needs for value creation; these collaborations can yield outcomes that are nonreplicable by competitor companies [6]. Based on the work of Lee et al. [6], this study develops the idea of “green co-innovation performance,” which focuses on co-innovation in the context of sustainability. Most studies have focused on how open innovation influences a company’s performance [11], whereas few have examined how open innovation operates in the context of sustainable development. However, through the exchange of ideas between customers, suppliers, and partners, a company can generate value [18]. Based on the preceding discussion, the following hypothesis is proposed:

**Hypothesis** (**H3**)**.** *Green open innovation activities are positively associated with green co-innovation performance.*

#### 2.2.3. Perceived Similarity has a Positive Effect on Green Open Innovation Activities and Green Co-Innovation Performance

Perceived similarity between a company and its partners is a key driver of innovation performance [19]. Perceived similarity can be categorized either as work style, which entails similar communication and interaction work habits, or as social category, which encompasses similar features such as nationality, culture, and ethnicity [9].

In open innovation, a similar work style is positively associated with an organization’s members’ willingness to consider their coworkers’ views [20]. Conversely, a lack of similarity in work style causes conflict and decreases commitment between a company and its partners. If two companies have similar work habits and communication styles, they tend to be highly attracted to each other, which facilities greater integration of knowledge. Both survival and growth of a company can be promoted through support from an external partner. A company can achieve green innovations through learning from an external partner. Moreover, when employees of companies belong to similar social categories, this is readily apparent; such perceived similarity can enhance the willingness to exchange information [21]. Social category similarity reduces gaps associated with cultural dissimilarity and language barriers, which can enhance knowledge integration [22]. Consequently, companies can share ideas with related companies and work together to create innovations in the field of sustainable development.

Successful green co-innovations rely heavily on interaction with business partners [11,23,24]. According to the sociological concept of homophily, similar companies are more likely to associate with each other. If the focal company and its business partners have a similar work style or social categories, communication and relationship building are easier [19]. Therefore, the focal company can collaborate and share knowledge to execute green co-innovation projects.

Green open innovation activities and green co-innovation performance are enhanced through perceived similarity. First, outside sources, such as suppliers and customers, can contribute to a company’s environmental management knowledge base. Moreover, perceived similarity between the company and its outside sources can improve social identification and facilitate the assimilation of external ideas. Second, the inside-out process enables a company to externalize its knowledge and R&D results. Companies can commercialize their green technologies in new markets. Perceived similarity helps companies adopt green technologies that are being used in related or unrelated marketplaces. Last, in companies with perceived similarity, knowledge flow is greatly enhanced, which creates market opportunities and value for all partners. Thus, the following hypotheses are proposed:

**Hypothesis** (**H4**)**.** *Perceived similarity is positively associated with green open innovation activities.*

**Hypothesis** (**H5**)**.** *Perceived similarity is positively associated with green co-innovation performance.*

### 2.3. Research Framework

**Figure 1:**
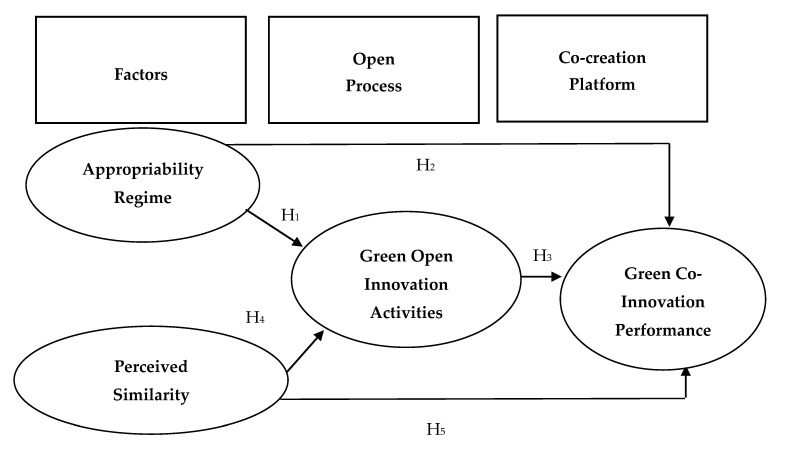
Research framework.

Previous studies have generally explored the topic of open innovation, but few studies have investigated a sustainable development view. This study adopted green open innovation activities as a mediator and explored the positive effects of an appropriability regime and perceived similarity on green co-innovation performance among Taiwanese manufacturing companies. Knowledge protection and sharing are typically in tension [7] because knowledge sharing may increase the competitiveness of competitors in the same industry [25]. This study aimed to determine the simultaneous effects of both factors, appropriability regime and perceived similarity, on green open innovation activities; furthermore, it investigated the mediation effect of green open innovation activities. Figure 1 displays a research framework that encompasses the findings of the literature on sustainable development, open innovation, and co-innovation performance.

## 3. Methodology, Measurement, and Data

### 3.1. Study Sample and Data Collection

This study focused on Taiwan’s manufacturing industry, which is a knowledge-intensive sector that adopts innovative ideas from diverse fields and turns them into new products. Most Taiwanese manufacturing companies are original equipment manufacturing (OEM) companies or original design manufacturing (ODM) companies. When pursuing green co-innovation, manufacturing companies require new knowledge beyond their conventional network of suppliers. Taiwanese industry is technologically dynamic and turbulent and relies on collaborative relationships among companies and their partners to develop new technologies. The unit of analysis is the business level, and the companies investigated are active in the information technology, electronics, and mechanical and electronic engineering fields.

The sample was randomly drawn from the 2018 Factory Operation Census prepared by Taiwan’s government published by the Ministry of Economic Affairs in Taiwan. Seven hundred questionnaires were sent to executives working in a range of departments, including environmental protection, marketing, manufacturing, purchasing, human resources, finance, and R&D departments. Research assistants called each sampled company to explain the objectives of the study and the content of the questionnaire. They further confirmed respondent names and job titles before mailing out the questionnaire. Research assistants also asked respondents to transfer the questionnaire to different department managers who belong to the same green project, and these questionnaires were completed. One questionnaire was answered for a single project in a company. This was done to maximize the response rate. Respondents were instructed to return the completed questionnaires within the weeks via mail. One hundred and ninety valid questionnaires were returned, an effective response rate of 27.14%.

To mitigate common method variance (CMV), respondents were required to answer various constructs presented in the questionnaire. Managers of R&D or manufacturing departments responded to “appropriability regime” questionnaires; managers of R&D or human resource departments responded to “perceived similarity” questionnaires; managers of marketing, manufacturing, or purchasing departments responded to “green open innovation activities” questionnaires; and CEOs or managers of environmental protection or R&D departments in Taiwanese companies responded to “green co-innovation performance” questionnaires. The anonymity and confidentiality of all responses were assured, and respondents were encouraged to be honest in their answers. Moreover, the researchers formally separated the antecedent, mediator, and consequence constructs; these all appeared to be unrelated [25].

This study utilizes Harman’s one-factor test to investigate the presence of common method variance (CMV). There are 17 items within the four constructs in this study. All the 17 items are entered into an exploratory factor analysis to determine the number of factors. If either a single factor emerges from the exploratory factor analysis, or one general factor accounts for the majority of the covariance among the 17 items, a problem of common method variance (CMV) appears. The result shows that there are four distinct factors with eigenvalues greater than 1.0, rather than a single factor. The four factors together also account for 80.63% of the total variance; the major factor constitutes 45.48% of the variance. Hence, there is no common method variance (CMV) problem in this study.

### 3.2. Measurement of Constructs

All items in this study were assessed using a 7-point Likert scale from 1 (*strongly disagree*) to 7 (*strongly agree*). The measurement of the constructs is described in the following text and refers to Table A1.

#### 3.2.1. Appropriability Regime

Companies must protect intellectual property through mechanisms such as copyright, patents, and trade secret designations. An appropriability regime can be effective for protecting knowledge from appropriation [7]. The “appropriability regime” construct included four items: (1) whether the company protects its innovations through patents; (2) whether the company protects its accomplishments through copyrights; (3) whether the company protects its accomplishments through trademarks; and (4) whether the company protects its accomplishments through long-term collaboration contracts [7].

#### 3.2.2. Perceived Similarity

The measurement of perceived similarity is divided into two parts: perceived work style similarity and perceived social category similarity, which are separately measured. Three items are used to measure perceived work style similarity: (1) whether the company and its partner companies share a similar work ethic; (2) whether the company and its partner companies have similar work habits; (3) whether the company and its partner companies have similar communication styles. Perceived social category similarity is measured by two items: (1) whether the company and its partner companies have similar personalities; (2) whether the company and its partner companies share a common cultural background [9,21].

#### 3.2.3. Green Open Innovation Activities

This study refers to Cheng and Huizingh [11], in order to develop an original construct termed “green open innovation activities. Green open innovation activities are measured through responses to four items: (1) our innovation projects entail the direct involvement of external partners (customers, competitors, academic institutions, consultants, suppliers, government entities); (2) our firm often sells licenses to patents, copyrights, or trademarks to other firms to capitalize on our innovation efforts; (3) our firm often offers royalty agreements to other firms to capitalize on our innovation efforts; (4) in innovation projects, our firm typically coordinates information exchange among partners [11,26].

#### 3.2.4. Green Co-Innovation Performance

This study refers to Lee et al. [6], in order to develop an original construct termed “green co-innovation performance.” The construct of green co-innovation performance is measured by four items: (1) whether the least environmentally impactful materials are selected by the firm and its partners during product design and development; (2) whether the selection of materials that consume the least amount of energy and resources is a paramount concern of the company and its partners during product design and development; (3) whether using the least amount of materials to produce a product is a paramount concern of the company and its collaborators; (4) whether reducing the consumption of water, coal, oil, and electricity during process development is a point of emphasis for the company and its collaborators [16,26].

## 4. Empirical Results

Structural equation modeling (SEM), which involves the analysis of measurement and structural models, is used to verify the hypotheses. The maximum likelihood estimation (MLE) method is executed in AMOS 17 to obtain empirical results. Appropriability regime and perceived similarity are antecedents in this study, green co-innovation performance is the consequent, and the mediator is green open innovation activities.

### 4.1. Results of the Measurement Model

Table 1, Table 2 and Table 3 present enterprise size, years established, companies’ means and standard deviations of the constructs. Positive correlations were identified among the appropriability regime, perceived similarity, green open innovation activities, and green co-innovation performance. Table 4 presents the result of factor analysis. Each construct in this study can be classified into only one factor. The measurement model exhibited acceptable levels of model fit (goodness of fit (GFI) = 0.894; comparative fit index (CFI) = 0.968; normed fit index (NFI) = 0.934; and root mean square error of approximation (RMSEA) = 0.067).

#### 4.1.1. Reliability

Several measures can be used to confirm measurement reliability. Evaluating the loadings of each construct’s items is one approach. Table 5 presents all the loadings (λ) of the constructs and further displays Cronbach’s α coefficients for the measure of reliability; 0.7 is generally considered the minimum acceptable value [27]. Here, the measurement was acceptably reliable because the Cronbach’s α of all constructs was greater than 0.7. In addition to reliability, the validity of the measurement tool is critical; this can be verified by evaluating content, discriminant, and convergent validity.

#### 4.1.2. Content Validity

The scale’s content validity was assessed by several measures. First, the constructs were established based on an extensive literature review concerning appropriability regime, perceived similarity, open innovation, and co-innovation performance. Questionnaire items were then defined following those in the literature. Moreover, innovation scholars were asked to review and modify the questionnaire items in the first pretest; only after completing this step were the questionnaires mailed to prospective respondents, who included 14 executive-level managers in manufacturing, marketing, human resources, purchasing, or R&D departments in various Taiwanese manufacturing companies. The 14 respondents were considered key informants capable of providing insight into the perceptions of all the constructs. These respondents completed the questionnaires and identified ambiguity in terms of meanings and other problems in the second pretest. Thus, content validity was established.

#### 4.1.3. Discriminant Validity

To assess the discriminative validity of the measurement, this study utilized Fornell and Larcker’s [28] measure, average variance extracted (AVE). Compared with the correlations between the construct and other constructs in the research framework, the square root of a construct’s AVE must be higher. For example, the AVE square roots of appropriability regime and perceived similarity were 0.811 and 0.846, respectively (Table 5), which were higher than the correlation between them (0.358; Table 3). Thus, discriminant validity was adequate between these constructs. As evident in Table 3, for all constructs the square roots of their AVEs were higher than the correlations among them (Table 3). Thus, for the measurement discussed herein, discriminant validity was acceptable.

#### 4.1.4. Convergent Validity

In Table 5, the AVEs of all constructs exceeded 0.5, indicating acceptable convergent validity. Thus, overall, the study’s measurements were adequately reliable and valid.

### 4.2. Results of the Structural Model

**Figure 2:**
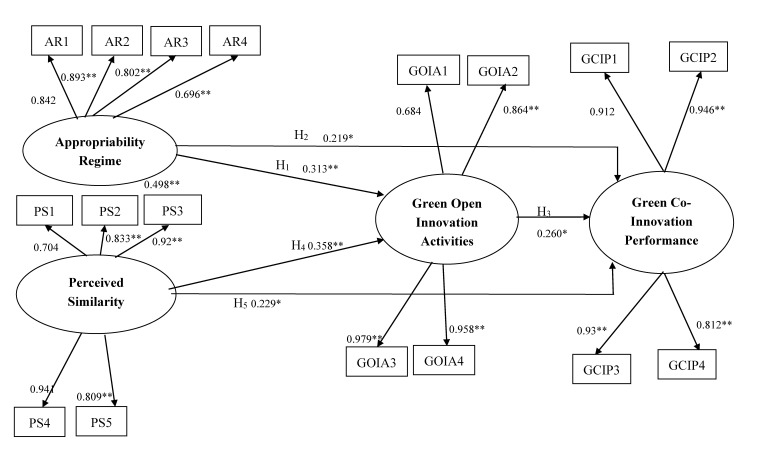
Full model results. Note: goodness of fit (GFI) = 0.901; normed fit index (NFI) = 0.939; root mean square error of approximation (RMSEA) = 0.062. * *p* < 0.05, ** *p* < 0.01.

Table 6 and Figure 2 present the results of SEM. According to the fit indices, the measures of model fit were acceptable (χ^2^ = 189.1, degrees of freedom (d.f.) = 110, χ^2^/d.f. = 1.71, GFI = 0.901, NFI = 0.939, and RMSEA = 0.062). The fit indices of the measurement and structural models were acceptable; to add or delete any path to or from the research framework would fail to significantly improve the fit. Additionally, the residuals of the covariance were small and centered near 0. Figure 2 displays the results of the complete model. All paths are presented in Table 6. Hypotheses H_1_, H_2_, H_3_, H_4_, and H_5_ were supported in the research model.

### 4.3. In-Depth Exploration of Data

Table 7 shows the duration of green projects and number of years companies had been established. Table 8 shows the results of *t* test. The *t* test was applied to confirm differences among groups. Depending on the duration of green project implementation, manufacturing companies were classified into two groups: “green project duration of three years or more” and “green project duration of fewer than three years.” During 2015, the Financial Supervisory Commission in Taiwan stated that listed companies have to compile corporate social responsibility reports. These companies should adopt internationally accepted guidelines in the compilation of corporate social responsibility (CSR) Reports. Such guidelines provide indicators in various dimensions, such as environment, society, and corporate governance. Therefore, a large number of Taiwanese manufacturing companies have devoted themselves to green practices in the past three years. The sample size of the “green project duration of three years or more” group was 101 and that of the “green project duration of fewer than three years” group was 89. According to the *t* tests, the degrees of green open innovation activity and green co-innovation performance for the “green project duration of three years or more” group were significantly higher than those for the “green project duration of fewer than three years” group in Table 7.

## 5. Conclusions

SEM was adopted to investigate the positive effects of appropriability regime and perceived similarity on green co-innovation performance in the Taiwanese manufacturing industry when considering green open innovation activities as the mediator. The results indicate that all the proposed hypotheses are supported.

Three implications emerge from this study. First, this study shifts the research focus from green innovation to green open innovation activities, which can increase resource productivity and give companies a competitive edge. Most Taiwanese manufacturing companies are original equipment manufacturing (OEM) firms, which make products according to the purchasing company’s specifications, or original design manufacturing (ODM) companies, which design and manufacture a product that is eventually sold under another company’s brand name. In the case of OEM companies, the purchasing companies have a major influence on the OEM company; if an OEM company is similar to its partners in work style or social category, they may be able to jointly pursue successful green open innovation activities. For ODM firms, corporate social responsibility can be a means to a competitive edge— green open innovation activities can create a leadership position in environmental management, which translates into a competitive advantage not readily imitated by competitors [29]. Therefore, perceived similarity can successfully enhance green open innovation activities. Under the trend of environmentalism, companies try to find green opportunities and realize the goal of sustainable development in their organizational operation. According to the results in this study, if companies want to increase their green open innovation activities, they must protect their innovations through patents and find partners with similarity. In the Chinese context, similar companies are more likely to associate with each other. If the focal company and its business partners have a similar work style or social categories, they can create value through the creation of joint value processes. Moreover, using stakeholder theory, future studies can emphasis relationships and engagement in mutually beneficial processes.

A second major implication is that a green project of longer duration translates into stronger green open innovation activities and green co-innovation performance. Degrees of green open innovation activity and green co-innovation performance for the “green project duration of three years or longer” group were higher than those for the “green project duration of fewer than three years” group. For long-term green projects, manufacturing companies in Taiwan are likely to exhibit strong green open innovation activities and green co-innovation performance. Eco-friendliness represents beliefs and values regarding norms of environmental stewardship and management. Many managers argue that green behavior and corporate performance necessarily conflict. However, companies that invest resources in accelerating green performance can improve the value of products, which can offset the added cost of eco-friendliness. As suggested by Porter and van der Linde [28], green strategies can provide a solution that benefits both the environment and a firm’s revenue. Growing environmental awareness has led many companies to act more socially responsibly, which has resulted in efforts to enhance green open innovation activities and green co-innovation performance through long-term sustainable projects. Moreover, future studies can apply institutional theory and explain the process of isomorphism in manufacturing industry.

The third major implication is that an appropriability regime and perceived similarity are critical antecedents to green open innovation activities. In Taiwan, 97.73% of manufacturing companies are small and medium-sized enterprises, most of which have insufficient resources to develop new products. For them, it is critical to protect their innovations through patents and copyrights to secure a competitive advantage. Taiwanese manufacturing and related companies have similar communication styles, which can align company and partner requirements for a high degree of green open innovation activities. Moreover, perceived similarity creates a harmonious working environment, which facilitates the operation of an eco-friendly business enterprise in a Chinese context, such as in Taiwan. Therefore, appropriability regimes and perceived similarity simultaneously determine a company’s green practices. Future studies can focus on green practices in Taiwanese small and medium-sized enterprises.

This study has four limitations. First, only manufacturing industry was sampled; a future research direction would be to examine other industries. Second, this study was restricted to Taiwan. To confirm the generalizability of the hypotheses in other cultural contexts, future research could select other countries and compare the results obtained with those of this study. Third, this study measured the appropriability regime construct by self-reported data. If future studies wish to measure this construct more precisely, second-hand data, such as government databases or bulletins, can be utilized. Fourth, there is a need to include timing-related measures of green open innovation activities and green co-innovation performance. Accordingly, longitudinal research could help to identify results in various developmental stages. The hypotheses in this study were confirmed through a questionnaire survey, which yielded only cross-sectional data. Therefore, no longitudinal data are available for analyzing dynamic changes in the constructs used to study Taiwanese companies. Nevertheless, this study discerned useful insights that can be the basis for future research.

## Figures and Tables

**Table 1:** Enterprise size.

Enterprise Size	Frequency	Percentage of Frequency
Small and medium enterprise (does not exceed 200 persons)	157	82.63%
Large enterprises (200 persons above)	33	17.37%

**Table 2:** Established years of companies.

Companies Established Years	Less Than 5 Years	Above 5 Years, Less Than 10 Years	Above10 Years, Less Than 15 Years	Above 15 Years, Less Than 20 Years	Above 21 Years, Less Than 25 Years	Above 25 Years, Less Than 30 Years	30 Years Above
Frequency	8	8	17	31	27	28	71
Percentage of Frequency	4.2	4.2	8.9	16.3	14.2	14.7	37.4

**Table 3:** Means, standard deviations, and correlations of the constructs.

Constructs	Mean	Standard Deviation	A	B	C	D
A. Appropriability regime	5.078	1.338				
B. Perceived similarity	5.041	1.125	0.358 **			
C. Green open innovation activities	4.303	1.307	0.432 **	0.456 **		
D. Green co-innovation performance	5.541	1.100	0.395 **	0.458 **	0.421 **	

Note: ** *p* < 0.01.

**Table 4:** Factor analysis.

Constructs	Number of Items	Number of Factors	Accumulation Percentage of Explained Variance
A. Appropriability regime	4	1	74.124%
B. Perceived similarity	5	1	77.794%
C. Green open innovation activities	4	1	83.607%
D. Green co-innovation performance	4	1	84.573%

**Table 5:** Loadings (λ) of items and Cronbach’s α coefficients and average variance extracted (AVE) of constructs.

Constructs	Items	λ	Cronbach’s α	AVE	The Square Root of AVE
A. Appropriability regime	AR1	0.842	0.883	0.659	0.811
	AR2	0.893 **			
	AR3	0.802 **			
	AR4	0.696 **			
B. Perceived similarity	PS1	0.704	0.928	0.715	0.846
	PS2	0.833 **			
	PS3	0.920 **			
	PS4	0.941 **			
	PS5	0.809 **			
C. Green open innovation activities	GOIA1	0.684	0.933	0.773	0.879
	GOIA2	0.864 **			
	GOIA3	0.979 **			
	GOIA4	0.958 **			
D. Green co-innovation performance	GCIP1	0.912	0.939	0.813	0.902
	GCIP2	0.946 **			
	GCIP3	0.930 **			
	GCIP4	0.812 **			

Note: ** *p* < 0.01.

**Table 6:** Path coefficient results.

Hypothesis	Results	Path Coefficient
H_1_	H_1_ is supported	0.313 **
H_2_	H_2_ is supported	0.219 *
H_3_	H_3_ is supported	0.260 *
H_4_	H_4_ is supported	0.358 **
H_5_	H_5_ is supported	0.229 *

Note: * *p* < 0.05, ** *p* < 0.01.

**Table 7:** Green projects’ duration.

Green Projects Duration	Fewer Than 3 Years	Above 3 Years, Fewer Than 5 Years	Above 5 Years, Fewer Than 10 Years	Above 10 Years, Fewer Than 20 Years	Above 20 Years
Companies	89	40	32	23	6

**Table 8:** Differences between groups with various green project durations.

Construct	Mean of “3 Years and Above in Green Project Duration” Group (A)	Mean of “Less Than 3 Years in Green Project Duration” Group (B)	A−B	Results
Green open innovation activities	4.495	4.084	0.411 * (2.167)	A > B
Green co-innovation performance	5.777	5.273	0.504 ** (3.233)	A > B

Note: * *p* < 0.05, ** *p* < 0.01. The number in the bracket is the *t* value.

## Appendix A

**Table A1:** The information on the questionnaire.

Construct	Items		Cronbach’s α	Resources
	Numbers	Content		
A. Appropriability regime	AR1	whether the company protects its innovations through patents	0.883	[7]
	AR2	whether the company protects its accomplishment through copyrights		
	AR3	whether the company protects its accomplishment through trademarks		
	AR4	whether the company protects its accomplishment through long-term collaboration contracts		
B. Perceived similarity	PS1	whether the company and its partner companies share a similar work ethic	0.928	[9,21]
	PS2	whether the company and its partner companies have similar work habits		
	PS3	whether the company and its partner companies have similar communication styles		
	PS4	whether the company and its partner companies have similar personalities		
	PS5	whether the company and its partner companies share a common cultural background		
C. Green open innovation activities	GOIA1	our innovation projects entail the direct involvement of external partners	0.933	[11,26]
	GOIA2	our firm often sells licenses to patents, copyrights, or trademarks to other firms to capitalize on our innovation efforts		
	GOIA3	our firm often offers royalty agreements to other firms to capitalize on our innovation efforts		
	GOIA4	in innovation projects, our firm typically coordinates information exchange among partners in our innovation projects		
D. Green co-innovation performance	GCIP1	whether the least environmentally impactful materials are selected by the firm and its partners during product design and development	0.939	[6,26]
	GCIP2	whether the selection of materials that consume the least amount of energy and resources is a paramount concern of the company and its partners during product design and development		
	GCIP3	whether using the least amount of materials to produce a product is a paramount concern of the company and its collaborators		
	GCIP4	whether reducing the consumption of water, coal, oil, and electricity during process development is a point of emphasis for the company and its collaborators		
